# Supplementary material for: Collaborative intelligence in AI: Evaluating the performance of a council of AIs on the USMLE
Source: PLOS Digit Health. 2025 Oct 9;4(10):e0000787. doi: 10.1371/journal.pdig.0000787 (PMC12510544; doi:10.1371/journal.pdig.0000787)
Supplement: S2 File — (ZIP) [file pdig.0000787.s002.zip › S2 Data Raw data files containing unprocessed question inputs and LLM outputs including transcripts of deliberations/STEP3_Question-22-1_Type-MC-NJ.docx]

**Facilitator** **[Round 1]:** A 27-year-old man comes to the office with his wife because of a recent episode of loss of muscle control. He says, ""I was at a reception, someone told a joke, and when I laughed, my legs collapsed!"" His wife says that he recovered in a few seconds and he did not lose consciousness. He has a long history of sleepiness and he is able to go to sleep quickly. He usually awakens feeling refreshed after a short nap. He has no history of similar episodes or hallucinations. There is no family history of similar problems. Vital signs are normal. Physical examination shows no abnormalities. Which of the following is the most likely diagnosis? (A) Narcolepsy (B) Primary hypersomnia (C) A seizure disorder (D) Sleep paralysis (E) Vasovagal syndrome

**AI_A:** Restating the question: A 27-year-old man experiences a sudden loss of muscle control during laughter. This phenomenon, where laughing causes muscle weakness, is usually instantaneous, and the individual recovers quickly without losing consciousness. He is also constantly sleepy but can quickly go to sleep and wakes up feeling refreshed from short naps. This man does not have any history of such episodes or hallucinations, and there's no record of family members with similar issues. His vital signs are normal and no abnormalities were found in his physical examination. We are asked to choose the most likely diagnosis from the following options: Narcolepsy, Primary hypersomnia, A Seizure disorder, Sleep paralysis or Vasovagal syndrome. Planning: The assumptions are that these episodes of muscle weakness (or temporary paralysis) are connected to his sleepiness and ability to fall asleep quickly. These episodes could be either due to a malfunction in the sleep-wake regulation (narcolepsy or primary hypersomnia), an episode of abnormal electrical activities in the brain (seizure disorder), or related to experiences connected to sleep and waking (sleep paralysis). Another assumption could be that they are due to an abrupt drop in heart rate and blood pressure leading to fainting (vasovagal syndrome). Clinical priorities would be first to rule out acute and dangerous conditions like seizure disorders and then examine sleep related disorders. Clinical reasoning: The absence of post-ictal confusion, and normal physical and neurological examination results make a seizure disorder less likely. Vasovagal syndrome usually presents with episodes of syncope but there's no loss of consciousness in this patient's case, making it less likely as well. While both narcolepsy and primary hypersomnia present with excessive sleepiness, cataplexy (sudden loss of muscle tone triggered by strong emotions such as laughter) is highly suggestive of narcolepsy. Sleep paralysis usually occurs upon falling asleep or waking and involves inability to move, not collapse due to muscle weakness, making it less appropriate in this context. Concluding: Considering the patient's excessive sleepiness, quick onset of sleep, refreshing short naps, and most importantly, episodes of sudden muscle weakness during laughter (cataplexy), narcolepsy seems to be the most probable diagnosis. Response: The most likely diagnosis in this scenario would be (A) Narcolepsy.

**AI_C:** RESTATING THE QUESTION: This is a scenario in which a 27-year-old man is experiencing unusual events, where laughter induces a sudden temporary loss of muscle control causing his legs to collapse, along with a long history of sleepiness and quick onsets of sleep from which he wakes up refreshed after short periods. There are no signs of physical abnormality, hallucinations, or related family health issues. The question seeks the most likely cause of these symptoms from the options provided: Narcolepsy, Primary Hypersomnia, a Seizure Disorder, Sleep Paralysis, and Vasovagal Syndrome. PLANNING: The question hints at signifiers of a potential sleep disorder. An assumption is that the man's condition is related to his sleep pattern, considering his history of excessive sleepiness. Let's question that assumption: it could also be related to neurological events or reflex due to emotional triggers like laughter. Evaluating these assumptions, it's clinically interesting to focus more on the connection between the sudden loss of muscle control and the sleepiness. These are events where the activating systems in the brain that keep us awake are overpowered by the brain's sleep-regulating mechanisms. CLINICAL REASONING: Firstly, Primary Hypersomnia, a Seizure Disorder, Sleep Paralysis, and Vasovagal Syndrome don't typically involve laughter-induced muscle collapses (termed cataplexy). Cataplexy is most commonly linked to narcolepsy, a neurological disorder that affects the control of sleep and wakefulness. Symptoms of narcolepsy include excessive daytime sleepiness, which can occur at any time, like while talking or driving, aligning with the man's story. Furthermore, the "sleep attacks" in narcolepsy can be refreshing, which matches his tendency to wake up feeling refreshed after a short nap. CONCLUDING: Following this line of clinical reasoning, the connective links between the patient's description of his symptoms, their onset, and their manifestation in the context of laughter and sleep led us down a distinct diagnostic route pointing to a specific sleep disorder. This case scenario seems characteristically consistent with the symptoms associated with narcolepsy, involving abrupt intrusion of REM sleep fragments into wakefulness, as shown by the sudden muscle weakness 'cataplexy' triggered by strong emotions like laughter and the excessive daytime sleepiness. RESPONSE: Therefore, based on the clinical reasonings and the pattern of symptoms described, the most likely diagnosis for the man's condition is (A) Narcolepsy.

**AI_B:** 1) RESTATING THE QUESTION: The clinical case in question discusses a 27-year-old male patient who experienced an episode where he lost control of his muscles after he laughed, causing his legs to collapse beneath him. Subsequently, he quickly recovered from the episode while maintaining consciousness. The patient has a history of becoming sleepy easily and quickly falling asleep, yet feels refreshed after short rests. No history of similar episodes, hallucinations or familial history of similar problems is reported. Physical examination and vital signs reveal no abnormalities. The aim is to identify the most probable diagnosis from the following alternatives: narcolepsy, primary hypersomnia, a seizure disorder, sleep paralysis, or vasovagal syndrome. 2) PLANNING: We should note the patient's rapid recovery, preservation of consciousness throughout the event, and refreshed feeling after short naps while considering the possible diagnoses. It is necessary to determine whether the symptoms indicate a sleep disorder or a neurological condition. Postulating that the patient's condition is narcolepsy could explain both the sleepiness and the muscle weakness triggered by laughter, known as cataplexy. Conversely, if the condition were primary hypersomnia, a seizure disorder, sleep paralysis, or vasovagal syndrome, we would need to account for the features of the episode and the patient's other symptoms separately. 3) CLINICAL REASONING: Narcolepsy is characterized by excessive daytime sleepiness and abnormal REM sleep manifestations, which include cataplexy, sleep paralysis, and hypnagogic hallucinations. Cataplexy, a sudden, brief loss of voluntary muscle tone triggered by emotions such as laughter, matches the described episode well. Patients with narcolepsy often fall asleep easily and feel refreshed after a short nap. On the other hand, primary hypersomnia involves long sleep periods but does not include anomalous REM sleep phenomena like cataplexy. A seizure disorder usually involves altered consciousness and post-ictal confusion, which the patient did not have. Sleep paralysis limits movement upon falling asleep or waking, rather than following laughter. And vasovagal syndrome results from an abrupt decrease in heart rate and blood pressure, leading to fainting; however, the patient's muscle weakness was laughter-induced and without loss of consciousness. 4) CONCLUDING: After a careful clinical thought process, the symptomatology and the sequence of symptoms point towards the diagnosis of narcolepsy. The patient's rapid onset of sleep, refreshment after quick naps, and importantly, the loss of muscle control post laughter (cataplexy) are classic symptoms of narcolepsy. 5) RESPONSE: Therefore, in this case, the most likely diagnosis for the 27-year-old man who experienced an episode of loss of muscle control after laughing, long history of sleepiness, and recovered refreshingly after short naps, is narcolepsy (Choice A).

**AI_E:** RESTATING THE QUESTION: The scenario describes a young man experiencing an episode of loss of muscle control following laughter, without losing consciousness. He suffers from chronic sleepiness but experiences refreshment after short naps. His medical background lacks prior similar episodes, hallucinations, or related familial issues, and physical examinations do not reveal any abnormalities. The question is asking me to determine the most likely diagnosis from among the options given, namely, narcolepsy, primary hypersomnia, a seizure disorder, sleep paralysis, and vasovagal syndrome. PLANNING: This scenario suggests that his symptoms are neurological rather than cardiac or psychiatric. Each option's assumption could be questioned: (A) Is the loss of muscle control due to narcolepsy, a neurological disorder characterized by excessive daytime sleepiness and other symptomatology such as cataplexy (a sudden and transient episode of muscle weakness accompanied by full conscious awareness)? (B) Could it be primary hypersomnia, another neurological disorder marked by excessive daytime sleepiness, but without cataplexy or other specific symptoms associated with narcolepsy? (C) Is it a seizure disorder, where sudden uncontrolled electrical disturbances in the brain cause changes in behavior, movements, feelings, or levels of consciousness? (D) Can it constitute sleep paralysis, a phenomenon in which an individual is unable to move or speak when falling asleep or waking up? (E) Could it be a vasovagal syncope, a reflex fainting response triggered by certain stimuli leading to a drop in heart rate and blood pressure, typically characterised by a brief loss of consciousness? CLINICAL REASONING: The narrative describes the patient experiencing an abrupt loss of muscle control after laughter—a symptom known as cataplexy, predominantly associated with narcolepsy. Narcolepsy also includes excessive sleepiness, which aligns with the patient's recurrent sleepiness, and the ability to obtain refreshing sleep after a short nap. Primary hypersomnia is a less likely answer because, while it encompasses excessive sleepiness, it typically does not induce cataplexy. Likewise, the lack of altered consciousness during the episode makes a seizure disorder improbable. Sleep paralysis is distinguishable as it occurs when transitioning between sleep and wakefulness without any triggers, such as laughter in this situation. Vasovagal syndrome usually involves a transient loss of consciousness, which was not reported in this patient's comments. CONCLUDING: Analyzing the information, the man's abrupt muscle weakness due to laughter (cataplexy), prolong and asynchronous sleep patterns, and rapid sleep onset all suggest narcolepsy. The presence of cataplexy is particularly suggestive as this is a defining characteristic of the disorder, and it's triggered by strong emotions such as laughter. Aside from narcolepsy, other options do not completely account for the patient's symptoms. RESPONSE: The most likely diagnosis for this patient based on his symptoms and details in the history, is (A) Narcolepsy.

**AI_D:** RESTATING THE QUESTION: This question is about a 27-year-old male who experienced a sudden loss of muscle control when he laughed, leading to a collapse, but rapidly recovered without losing consciousness. He has chronic sleepiness and can easily nap, even for short durations, from which he usually wakes refreshed. He has not had similar episodes before, nor does he hallucinate. His family history is unremarkable for similar incidents. His vitals and physical examination are within normal limits. We are required to identify the most likely diagnosis from the list provided: (A) Narcolepsy (B) Primary hypersomnia (C) A seizure disorder (D) Sleep paralysis (E) Vasovagal syndrome PLANNING: To answer this question, we must consider the man's clinical presentation and cross-match it with each possible diagnosis. We'll define each condition and identify their key features: (A) Narcolepsy: A neurological disorder that causes excessive daytime sleepiness and uncontrollable sleep attacks. Other symptoms can include cataplexy (weakness or paralysis triggered by strong emotions), sleep paralysis, and hypnagogic hallucinations. (B) Primary hypersomnia: Characterized by excessive daytime sleepiness (EDS) without narcoleptic symptoms (like cataplexy, paralysis, hallucination). (C) A seizure disorder (epilepsy): Results in recurrent seizures due to abnormal electrical activity in the brain. These can result in loss of consciousness and muscle spasms but are typically not triggered by specific emotions or actions and are not usually associated with excessive sleepiness. (D) Sleep paralysis: A phenomenon where a person temporarily experiences an inability to move or speak while falling asleep or waking up. It's typically associated with vivid hallucinations. (E) Vasovagal syncope: A sudden drop in heart rate and blood pressure leading to fainting, often in response to a stressful trigger. CLINICAL REASONING: The patient's main symptoms are excessive daytime sleepiness, quick initiation of sleep, and short refreshing naps, which all indicate a potential sleep disorder. The sudden loss of muscle control caused by laughter points towards cataplexy, a characteristic symptom of narcolepsy. Instances of cataplexy are usually brief and the person remains conscious throughout. Narcolepsy would also explain the excessive sleepiness and the ability to sleep quickly. Although the patient doesn't present with sleep paralysis or hallucinations, multiple symptoms may not always be present in every individual patient. CONCLUSION: Comparing the patient's symptoms with the definitions and key features of the conditions given as options, it's clear that, while several conditions could explain parts of the presentation (e.g., primary hypersomnia for excessive sleepiness and sleep paralysis for temporary inability to move), narcolepsy is the only condition that accounts for both the sleepiness and the episode of muscle loss triggered by laughter without loss of consciousness. RESPONSE: The most likely diagnosis for the patient's condition is (A) Narcolepsy.

**Facilitator** The consensus statement synthesizes the responses of AI_A, AI_B, AI_C, AI_D, and AI_E as follows: The case presents a 27-year-old man who experiences sudden loss of muscle control during laughter, a phenomenon known as cataplexy. This is usually instantaneous, and the individual recovers quickly without losing consciousness. He also exhibits chronic sleepiness but can quickly fall asleep and wakes up feeling refreshed from short naps. There is no history of similar episodes, hallucinations, or family members with similar issues. His vital signs are normal, and no abnormalities were found in his physical examination. The potential diagnoses to consider include Narcolepsy, Primary hypersomnia, a Seizure disorder, Sleep paralysis, and Vasovagal syndrome. The clinical reasoning process involves ruling out conditions that do not align with the patient's symptoms. A seizure disorder is less likely due to the absence of post-ictal confusion and normal physical and neurological examination results. Vasovagal syndrome typically presents with episodes of syncope, but the patient does not lose consciousness, making it less likely. Sleep paralysis usually occurs upon falling asleep or waking and involves an inability to move, not collapse due to muscle weakness. Primary hypersomnia involves long sleep periods but does not include anomalous REM sleep phenomena like cataplexy. The patient's symptoms of excessive sleepiness, quick onset of sleep, refreshing short naps, and most importantly, episodes of sudden muscle weakness during laughter (cataplexy), are highly suggestive of narcolepsy. Narcolepsy is a neurological disorder that affects the control of sleep and wakefulness, characterized by excessive daytime sleepiness and abnormal REM sleep manifestations, which include cataplexy, sleep paralysis, and hypnagogic hallucinations. Therefore, based on the clinical reasoning and the pattern of symptoms described, the most likely diagnosis for the man's condition is Narcolepsy.
